# Level and potential social-ecological factors associated with physical inactivity and sedentary behavior among Moroccan school-age adolescents: a cross-sectional study

**DOI:** 10.1186/s12199-017-0657-0

**Published:** 2017-05-18

**Authors:** Abdelghaffar El-ammari, Hicham El kazdouh, Siham Bouftini, Samira El fakir, Youness El achhab

**Affiliations:** 1 Laboratory of Epidemiology, Clinical Research and Community Health, Faculty of Medicine and Pharmacy, University of Sidi Mohamed Ben Abdellah, Fez, Morocco; 2 Regional Centre for Careers Education and Training, Taza, Morocco

**Keywords:** Physical inactivity, Sedentary behavior, Adolescents, Associated factors, Morocco

## Abstract

**Background:**

Creating a successful intervention that supports an active lifestyle and prevents sedentary one requires a better understanding of the factors associated with physical inactivity (PI) and sedentary behavior (SB). However, these factors have not been assessed among Moroccan adolescents. This study aimed to determine prevalence of PI and SB and to explore their potential social-ecological associated factors in school-age adolescents.

**Methods:**

In this cross-sectional study, 764 students (age range, 14–19 years) were enrolled from six schools in Taza city, Morocco. The Global School-based Student Health Survey was used to collect data about variables. We used bivariate and multivariate analyses to assess relations between dependent and independent variables.

**Results:**

Overall, the prevalence of PI was 79.5% and SB was 36.5%. Among girls, these rates were higher (87.0 and 39.1%, respectively) than rates shown in boys (70.9 and 33.6%, respectively). In the multivariate logistic regression analysis, PI was associated with the following variables: illiterate father, hunger, suicidal ideation, inadequate vegetable consumption, and absence from physical education classes. Age, inadequate vegetable consumption, and absenteeism were associated with SB.

**Conclusions:**

The prevalence of PI and SB is high, especially among girls. Thus, there is an urgent need to implement appropriate interventions to reduce PI and SB levels in secondary school-age adolescents and the associated factors identified can be useful.

**Electronic supplementary material:**

The online version of this article (doi:10.1186/s12199-017-0657-0) contains supplementary material, which is available to authorized users.

## Background

Adolescence is a critical stage in human development, during which children undergo hormonal changes and brain maturation and acquire a new set of health-related behaviors that are likely to persist into adulthood [1, 2]. Although most adolescents are perceived as healthy, important risk factors for disease, such as physical inactivity (PI) and sedentary behavior (SB), emerge during this period of life [3, 4].

PI is a global public health concern among adolescents in high-income countries and increasingly in low-and middle-income countries (LMIC) [5]. According to the World Health Organization (WHO), to be healthy, adolescents should accumulate at least 60 min of moderate- to vigorous-intensity physical activity daily [6]. Despite the established health benefits [6, 7], more than 80% of adolescents worldwide do not reach this threshold [6].

SB is another major concern among adolescents and is defined as spending three or more hours per day sitting (excluding time sitting in school or doing homework) [8, 9]. It refers to activities that require low levels of energy expenditure (e.g., sitting, watching TV, and engagement in other screen-based activities, including driving) [8, 9]. A recent systematic review found that adolescents spend 57% of their time after school in sedentary activities [10].

In our approach, as in many other studies [11–13], PI and SB are regarded as different constructs because they contribute independently to adverse health outcomes [14]. That is, even highly active individuals are susceptible to the negative health effects of excess sedentary activities. Excess PI and SB in adolescents can lead to several negative health outcomes, including increased adiposity, lower fitness, increased cardiometabolic risk, and poor self-esteem [7, 15, 16]. PI can contribute to several noncommunicable diseases such as heart disease, diabetes, and cancer [17]. In 2008, more than 5.3 million of the 57 million deaths that occurred worldwide were attributable to PI.

Therefore, interventions to change health behaviors and improve health outcomes should focus on decreasing both PI and SB in adolescents. Creating a successful intervention that supports an active lifestyle and prevents a sedentary one requires a better understanding of the factors associated with PI and SB [11, 18]. Currently, there is strong evidence that theories of behavioral sciences can be used to understanding the associated factors of physical activity [19]. Because physical activity in adolescents is a complex behavior affected by a vast array of factors, ecological models are often advocated [11, 19–24]. These models incorporate a broad view of factors influencing physical activity and the interactions between them. These factors range from individual to social and physical environment characteristics [21].

Numerous groups have sought to assess the factors associated with PI and/or SB among adolescents, although these studies have been often conducted in high-income countries. Generally, the consistent associated factors were mainly related to individual characteristics (including age, sex, socioeconomic status, and psychosocial health) or related to social and physical environment factors (including social support, opportunities to exercise, and community sports) [11, 13, 20, 22–30].

In Morocco, data on PI and SB in adolescents are scarce, although this country is experiencing an epidemiologic transition [31, 32]. Beginning in 2010, national data from the Global School-based Student Health Survey (GSHS) have reported high prevalence of PI (85.6%) and SB (30%) among Moroccan school-age adolescents (age range, 13–15 years) [33]. One local study in Kenitra examined behaviors of adolescents (15–19 years) and found roughly similar prevalence for SB but lower prevalence of PI [34]. Until now, there have been no publications in Morocco on the factors associated with PI or SB among adolescents, thus making it difficult to develop an effective intervention. Information that will emerge from this study can fill this gap. In this cross-sectional study, our aim was to determine the prevalence of PI and SB and to explore their potential social-ecological associated factors among secondary school-age adolescents (14–19 years) in Taza city, Morocco.

## Methods

### Sampling design and study population

We conducted a school-based cross-sectional study from February to March 2016 in Taza, a medium-sized city (207 984 inhabitants) [35] in north-central Morocco. This study is part of a macro project designed to investigate the health risk behaviors of adolescents with the goal of developing an intervention to improve their health [36]. The sample size was estimated using the Epi-info software based on a population of 30,000 students [37]. It was calculated based on the 95% confidence level, and the population proportion has been assumed to be 0.50, as this magnitude yield the maximum possible sample size required. A multistage stratified cluster random sampling method was used to select the sample, and a design effect coefficient of two was chosen resulting in a final sample size of 760 (380 × 2) students, both sexes included. The sampling frame was the roster of middle and high secondary schools for the whole Taza city (17 schools). Stratified cluster random sampling was used to produce more precision and better representatives of the study population. In the first stage, two schools were randomly selected with each three neighborhoods defined by socio-economic level (disadvantaged, average, and advantaged). At the second stage, four classes were randomly sampled in each selected school on the day of the survey. All students in the selected classes were eligible to participate in the study. Because all students in the selected classes were included, the sample size was larger (*n* = 800) than estimated (*n* = 760). Only students who answered all questions were considered, resulting in a sample of 764 adolescents.

### Data collection and variables

The GSHS questionnaire was designed to assess health behaviors among students aged 13 to 17 years. It was developed by the WHO in collaboration with other institutions: the US Centers for Disease Control and Prevention (CDC), United Nations Children’s Fund (UNICEF), United Nations Educational, Scientific and Cultural Organization (UNESCO), and United Nations Joint Programme on HIV/AIDS (UNAIDS) [38]. This tool has been used in more than 120 countries worldwide, including Morocco [38]. Students were approached and asked to complete the Moroccan version of the GSHS, together with a questionnaire that included sociodemographic questions; details and data of the GSHS are available at the GSHS sections of the WHO website [38]. The questionnaire delivery was done by a trained investigator with help from a school official during one regular class period at classroom.

We explored two dependent variables in this study: PI and SB. Perceived PI of adolescents was assessed by the following question: “During the past 7 days, on how many days were you physically active for a total of at least 60 min per day? Add up all the time you spent in any kind of physical activity each day.” Introductory statements to the question included definition of physical activity and country-specific examples of the activities (e.g., running, fast walking, biking, dancing, and football) and specified that physical education or gym class should not be included. Adolescents were qualified as physically inactive if they did not obtain at least 60 min of physical activity per day on at least 5 days per week. Response options ranged from 1 (0 days) to 8 (7 days), coded as 1 (0–4 days) and 0 (5–7 days).

Perceived SB of adolescents was assessed by the following question: “How much time do you spend during a typical or usual day sitting and watching television, playing computer games, talking with friends, or doing other sitting activities, such as Play Station or playing chess?” Introductory statements to the question indicated to not include time spent sitting in school or doing homework. Adolescents were considered sedentary if they spent three or more hours per day on these sitting activities. Response options ranged from 1 (less than 1 h per day) to 6 (more than 8 h per day), coded as 1 (three or more hours per day) and 0 (less than 3 h per day).

We placed responses from the health questionnaire into five categories: demographic and biological, psychological, behavioral, and social; these comprised our independent variables. This categorization, based on an ecological approach of health behavior, was proposed by Sallis et al. [18] and has been used by other studies [11, 13]. For more details on independent variables (see Additional file 1).

### Statistical analyses

Data were coded, entered, checked for completeness and inconsistencies, and analyzed using SPSS version 19 (SPSS, Inc., Chicago, IL, USA). Both bivariate and multivariate analyses were performed to establish the possible associations between independent variables and PI and SB (binary dependent variables) for boys and girls separately. Independent variables were entered in the final multivariate model if they had a significant association with a dependent variable in the bivariate analysis; only covariates that were statistically significant at *P* < 0.20 were included in the logistic-regression model. Statistical significance was defined at *P* < 0.05. Odds ratios with 95% confidence intervals were derived where appropriate.

### Ethics statement

School and parental consents as well as students’ approval for conducting the survey were obtained. Student privacy was protected through anonymous and voluntary participation. The study protocol was approved by the Faculty of Medicine and Pharmacy of Casablanca Research Ethics Committee and by the National Control Commission for the Protection of Personal Data (A-RS-193-2015). Provincial Directorate of Education Ministry in Taza had authorized this survey.

## Results

### Sample characteristics and prevalence of PI and SB

**Fig. 1:**
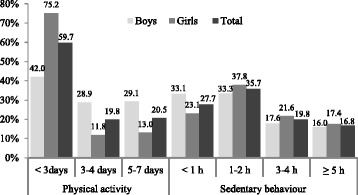
Prevalence and duration of physical activity per week and sedentary behavior per day among adolescents

The study population consisted of 764 (response rate of 95.5%) school-age adolescents (14–19 years old); 53.27% were girls. Overall, the prevalence of PI was 79.5% and SB was 36.5% (Fig. 1). We found that PI and SB occurred more often in girls (87.0 and 39.1%, respectively) than in boys (70.9 and 33.6%, respectively). Table 1 shows the numbers of boys and girls with PI and SB versus health behavior responses within the independent variable categories.

**Table 1:** PI and SB by independent variables

Independent variable	Total *n* (%)	Physical inactivity (<5 days/weeks)		Sedentary behavior (≥3 h or more)	
		Boys, no. (%)	Girls, no. (%)	Boys, no. (%)	Girls, no. (%)
Demographic/biological					
Age in years					
14–15	220 (28.8)	74 (77.9)	103 (82.4)	29 (30.5)	66 (52.8)
16–17	347 (45.4)	100 (65.4)	173 (89.2)	60 (39.2)	65 (33.5)
18–19	197 (25.8)	79 (72.5)	78 (88.6)	31 (28.4)	28 (31.8)
Education level of parents					
Illiterate mother (reference = educated)	286 (37.4)	106 (70.7)	120 (88.2)	39 (26.0)	44 (32.4)
Illiterate father (reference = educated)	102 (13.4)	38 (70.4)	38 (79.2)	13 (24.1)	14 (29.2)
Perceived family income					
Lower	76 (9.9)	31 (66.0)	26 (89.7)	9 (19.1)	11 (37.9)
Average	580 (75.9)	193 (72.8)	270 (85.7)	95 (35.8)	127 (40.3)
Higher	108 (14.1)	29 (64.4)	58 (92.1)	16 (35.6)	21 (33.3)
Being hunger (sometimes/mostly/always)	129 (16.9)	65 (84.4)	47 (90.4)	19 (24.7)	19 (36.5)
Psychological					
Loneliness (mostly/always)	180 (23.6)	43 (67.2)	103 (88.8)	19 (29.7)	57 (49.1)
Anxiety (mostly/always)	167 (21.9)	47 (65.3)	86 (90.5)	24 (33.3)	45 (47.4)
Suicidal ideation (reference = no)	120 (15.7)	27 (55.1)	62 (87.3)	23 (46.9)	38 (53.5)
No close friends (reference = 1 friend or more)	69 (9.0)	22 (71.0)	35 (92.1)	10 (32.3)	10 (26.3)
Behavioral					
Skipping breakfast (reference = sometimes/mostly/always)	162 (21.2)	40 (72.7)	91 (85.0)	20 (36.4)	53 (49.5)
Inadequate fruit consumption (<2 servings)	341 (44.6)	133 (71.9)	139 (89.1)	59 (31.9)	50 (32.1)
Inadequate vegetable consumption (<3 servings)	506 (66.2)	174 (77.0)	244 (87.1)	65 (28.8)	101 (36.1)
Not walking or biking to school (less than 5 days)	224 (29.3)	78 (75.0)	107 (89.2)	39 (37.5)	37 (30.8)
No attendance of physical education (2 or more times)	113 (14.8)	44 (84.6)	54 (88.5)	21 (40.4)	22 (36.1)
Physical fight (1 or more times)	261 (34.2)	128 (71.5)	72 (87.8)	70 (39.1)	46 (56.1)
Smoking (1 or more times during)	124 (16.2)	80 (72.7)	13 (92.9)	43 (39.1)	8 (57.1)
Alcohol/illicit drug use (reference = 0 time)	88 (11.5)	50 (65.8)	10 (83.3)	30 (39.5)	7 (58.3)
Absenteeism (reference = 0 day)	325 (42.5)	134 (71.3)	122 (89.1)	76 (40.4)	74 (54.0)
Social					
Lack of peer support in school (reference = mostly/always)	500 (65.4)	160 (70.8)	243 (88.7)	69 (30.5)	101 (36.9)
Lack of parental supervision (reference = mostly/always)	474 (62.0)	161 (71.6)	224 (90.0)	72 (32.0)	90 (36.1)
Lack of parental connectivity (reference = mostly/always)	572 (74.9)	194 (69.8)	255 (86.7)	96 (34.5)	120 (40.8)
Lack of parental bonding (reference = mostly/always)	511 (66.9)	184 (71.3)	226 (89.3)	86 (33.3)	93 (36.8)
Bullied (1 day or more)	176 (23.0)	19 (73.1)	132 (88.0)	8 (30.8)	73 (48.7)

### PI associated factors

We assessed associations between PI and perceived social-ecological factors using bivariate and multivariate regression analyses (Table 2). Among boys, in bivariate analysis, hunger, inadequate consumption of vegetables, and low attendance of physical education were positively associated with PI. Being in the 16- to 17-year age group and having suicidal ideations were negatively associated with PI. In multivariate analysis, a positive association with PI was found for hunger and inadequate vegetable consumption, with 16- to 17-year age group and suicidal ideation again being negative associations. Among girls, a lack of parental supervision was positively associated with PI in bivariate regression. In multivariate analysis, only education level of fathers (illiterate) was negatively associated with PI.

**Table 2:** Associations between physical inactivity prevalence and independent variables in adolescents by gender

Independent variable	Boys		Girls	
	UOR (95% CI)	AOR (95% CI)	UOR (95% CI)	AOR (95% CI)
Demographic/biological				
Age in years				
14–15	1.00	1.00	1.00	1.00
16–17	0.54 (0.30–0.96)*	0.54 (0.29–1.01)	1.76 (0.92–3.36)	1.56 (0.80–3.02)
18–19	0.75 (0.39–1.42)	0.51 (0.25–1.02)	1.67 (0.75–3.72)	1.60 (0.71–3.64)
Education level of parents				
Illiterate mother (reference = educated)	0.98 (0.62–1.56)		1.19 (0.63–2.22)	
Illiterate father (reference = educated)	0.97 (0.52–1.83)		0.52 (0.24–1.11)	0.41 (0.18–0.92)*
Perceived family income				
Lower	1.00		1.00	
Average	0.94 (0.40–2.21)		1.34 (0.30–6.02)	
Higher	1.38 (0.71–2.68)		0.69 (0.20–2.38)	
Being hunger (sometimes/mostly/always)	2.65 (1.36–5.15)**	4.13 (1.95–8.75)***	1.47 (0.56–3.88)	
Psychological				
Loneliness (mostly/always)	0.81 (0.45–1.45)		1.26 (0.65–2.46)	
Anxiety (mostly/always)	0.72 (0.42–1.25)		1.57 (0.74–3.35)	
Suicidal ideation (reference = no)	0.45 (0.24–0.83)**	0.35 (0.17–0.71)**	1.04 (0.48–2.24)	
No close friends (reference = 1 friend or more)	1.01 (0.45–2.26)		1.83 (0.54–6.17)	
Behavioral				
Skipping breakfast (reference = sometimes/mostly/always)	1.11 (0.59–2.12)		0.80 (0.43–1.51)	
Inadequate fruit consumption (<2 servings)	1.11 (0.70–1.75)		1.37 (0.74–2.53)	
Inadequate vegetable consumption (<3 servings)	2.20 (1.38–3.52)***	2.48 (1.51–4.08)***	1.05 (0.56–1.95)	
Not walking or biking to school (less than 5 days)	1.34 (0.80–2.24)		1.33 (0.69–2.59)	
No attendance of physical education (2 or more times)	2.53 (1.15–5.57)*	2.89 (1.27–6.57)**	1.18 (0.51–2.76)	
Physical fight (1 or more times)	1.06 (0.67–1.68)		1.10 (0.53–2.29)	
Tobacco use (1 or more times)	1.14 (0.69–1.88)		–	
Alcohol/illicit drug use (reference = 0 time)	0.74 (0.43–1.27)		–	
Absenteeism (reference = 0 day)	1.04 (0.66–1.65)		1.33 (0.71–2.52)	
Social				
Lack of peer support in school (reference = mostly/always)	0.99 (0.62–1.59)		1.55 (0.86–2.81)	1.41 (0.77–2.61)
Lack of parental/guardian supervision (reference = mostly/always)	1.09 (0.68–1.75)		1.93 (1.08–3.45)*	1.56 (0.84–2.92)
Lack of parental/guardian connectivity (reference = mostly/always)	0.78 (0.44–1.38)		0.93 (0.48–1.78)	
Lack of parental/guardian bonding (reference = mostly/always)	1.08 (0.65–1.79)		1.70 (0.95–3.04)	1.58 (0.84–2.97)
Bullied (1 day or more)	1.13 (0.46–2.76)		1.16 (0.63–2.12)	

*UOR* unadjusted odds ratio, *AOR* adjusted odds ratio, *CI* confidence interval

*P < 0.05; ** *P* ≤ 0.01; *** *P* ≤ 0.001

### SB associated factors

We also assessed associations between SB and perceived social-ecological factors using bivariate and multivariate regression analyses (Table 3). Among boys, a positive association with SB was found for high socioeconomic level, suicidal ideation, and absenteeism, whereas education level of mothers and inadequate consumption of vegetables were negatively associated with SB. In multivariate analysis, inadequate consumption of vegetables and absenteeism were negatively associated with SB. For girls, positive associations with SB were found for loneliness, suicidal ideation, skipping breakfast, physical fight, absenteeism, and bullying. Older adolescents, inadequate consumption of fruits, and deficiency of walking or biking to school were negatively associated with SB. In multivariate analysis, only older adolescents and absenteeism were associated with SB.

**Table 3:** Associations between sedentary behavior prevalence and independent variables in adolescents by gender

Independent variable	Boys		Girls	
	UOR (95% CI)	AOR (95% CI)	UOR (95% CI)	AOR (95% CI)
Demographic/biological				
Age in years				
14–15	1.00	1.00	1.00	1.00
16–17	1.47 (0.85–2.53)	1.60 (0.87–2.91)	0.45 (0.28–0.71)***	0.46 (0.27–0.77)**
18–19	0.91 (0.50–1.65)	1.10 (0.55–2.18)	0.42 (0.24–0.74)**	0.38 (0.20–0.72)**
Education level of parents				
Illiterate mother (reference = educated)	0.55 (0.35–0.87)**	0.72 (0.43–1.20)	0.65 (0.42–1.00)	0.93 (0.55–1.58)
Illiterate father (reference = educated)	0.58 (0.30–1.13)	0.74 (0.35–1.55)	0.61 (0.32–1.17)	0.70 (0.31–1.57)
Perceived family income				
Lower	1.00	1.00	1.00	
Average	2.33 (0.90–6.02)	2.03 (0.73–5.65)	0.82 (0.33–2.04)	
Higher	2.36 (1.09–5.09)*	1.82 (0.79–4.18)	1.11 (0.51–2.42)	
Being hunger (sometimes/mostly/always)	0.58 (0.33–1.03)	0.57 (0.30–1.10)	0.88 (0.48–1.62)	
Psychological				
Loneliness (mostly/always)	0.80 (0.45–1.45)		1.79 (1.16–2.77)**	1.42 (0.84–2.40)
Anxiety (mostly/always)	0.98 (0.57–1.70)		1.56 (0.98–2.49)	1.11 (0.64–1.93)
Suicidal ideation (reference = no)	1.92 (1.05–3.54)*	1.98 (0.96–4.06)	2.05 (1.22–3.43)**	1.22 (0.66–2.26)
No close friends (reference = 1 friend or more)	0.94 (0.43–2.06)		0.53 (0.25–1.12)	0.45 (0.20–1.03)
Behavioral				
Skipping breakfast (reference = sometimes/mostly/always)	1.15 (0.63–2.10)		1.80 (1.15–2.81)**	1.45 (0.88–2.40)
Inadequate of fruit consumption (<2 servings)	0.85 (0.55–1.32)		0.62 (0.40–0.93)*	0.79 (0.49–1.27)
Inadequate of vegetable consumption (<3 servings)	0.56 (0.36–0.88)*	0.56 (0.35–0.90)*	0.67 (0.44–1.03)	0.66 (0.41–1.07)
Not walking or biking to school (less than 5 days)	1.27 (0.79–2.05)		0.60 (0.38–0.95)*	0.70 (0.42–1.16)
No attendance of physical education (2 or more times)	1.41 (0.77–2.58)		0.86 (0.49–1.52)	
Physical fight (1 or more times)	1.64 (1.06–2.56)	1.49 (0.91–2.43)	2.40 (1.47–3.92)***	1.41 (0.79–2.51)
Tobacco use (1 or more times)	1.42 (0.89–2.26)	1.02 (0.60–1.74)	2.14 (0.73–6.28)	1.66 (0.47–5.87)
Alcohol/illicit drug use (reference = 0 time)	1.38 (0.82–2.34)		2.24 (0.70-7.18)	
Absenteeism (reference = 0 day)	1.93 (1.23–3.03)**	1.67 (1.02–2.73)*	2.56 (1.68–3.90)***	2.20 (1.35–3.60)**
Social				
Lack of peer support in school (reference = mostly/always)	0.69 (0.44–1.08)	0.71 (0.44–1.17)	0.76 (0.50–1.15)	0.80 (0.50–1.29)
Lack of parental/guardian supervision (reference = mostly or always)	0.82 (0.52–1.29)		0.73 (0.49–1.10)	0.75 (0.47–1.19)
Lack of parental/guardian connectivity (reference = mostly/always)	1.21 (0.71–2.07)		1.31 (0.83–2.06)	
Lack of parental/guardian bonding (reference = mostly/always)	0.96 (0.59–1.56)		0.78 (0.52–1.17)	
Bullied (1 day or more)	0.87 (0.37–2.06)		1.89 (1.25–2.85)**	1.50 (0.94–2.40)

*UOR* unadjusted odds ratio, *AOR* adjusted odds ratio *CI* confidence interval

*P < 0.05; **P ≤ 0.01; ***P ≤ 0.001

## Discussion

### Prevalence of PI and SB

The primary aim of our study was to determine the prevalence of PI and SB among secondary school-age adolescents in Morocco. We found PI and SB to be high among adolescents, at 79.5 and 36.5%, respectively. We also found that girls were physically more inactive (87.0%) than boys (70.9%) (*P* < 0.001) and more sedentary (39.1% versus 33.6%), although not significantly (*P* > 0.05).

Our results are similar to those from the WHO-GSHS survey conducted in Morocco in 2010 [38], which reported that the overall prevalence of PI was 82.6% (86.7% for girls and 79.2% for boys). In contrast, Hamrani et al. [34] reported that the overall prevalence of physically inactive adolescents was only 21%. This difference may be due to a different tool used to measure PI.

In studies that used the same measurement tool and that were conducted in LMIC, our results are generally consistent. Al Subhi et al. [39], in a cross-country study, demonstrated that the overall prevalence of PI was 81% in 10 eastern Mediterranean countries. In a meta-analysis study conducted in 65 LMIC, low physical activity was shown in 71.4% of adolescents [40]. In eight African countries, Peltzer et al. [26] found that 85.8% of school children were frequently physically inactive and that this was significantly higher among girls than boys. Furthermore, in 34 mainly developing countries, Guthold et al. [12] reported that only 23.8% of boys and 15.4% of girls met physical activity recommendations.

Regarding SB, we reported a high overall prevalence (36.5%), especially among girls. In the WHO-GSHS survey [38] conducted in Morocco in 2010, the overall prevalence of SB was 26.5% without significant difference by gender (*P* > 0.05). Our results are generally similar to other studies from LMIC that used the same measure of SB [12, 26, 32, 34, 39].

Urbanization and globalization may have contributed to the high levels of PI and sedentary lifestyle in LMIC countries [12]. In the Arab world, girls are more inactive than boys, perhaps due to the conservative social norms and cultural restrictions on outdoor activities and exercise for women [41]. Special intervention programs are needed for this subgroup.

### PI and SB associated factors

Our secondary aim was to identify social-ecological factors associated with PI and SB in adolescents. PI and SB were associated with following variables: demographic and biological variables (age, illiterate fathers, and hunger), psychological variables (suicidal ideation), and behavioral variables (inadequate vegetable consumption, no attendance of physical education, and absenteeism). Social variables were not associated with PI and SB. We also found that associated factors of PI were different from those of SB, confirming that they are independent entities. Furthermore, within each entity (PI and SB), the associated factors were different by gender. There is evidence that gender represents a consistent determinant of PI and SB [11, 13, 18, 20, 23].

The most frequently reported associated factors have been those within the demographic/biological category, with sex, age, and socioeconomic status having the highest impact [13, 20]. Unlike in previous studies [18, 20, 23], our study did not find significant associations between age and PI, which was also shown in other studies [11, 25, 42]. Generally, the prevalence of SB increases with increased age [20, 43]. However, we observed the prevalence of SB to have a net reduction with increased age among girls, perhaps explained by the fact that older girls help their mothers in household tasks, which in return reduces time spent watching TV or using other screen-based devices.

Socioeconomic status (measured here as perceived family income) was not significantly associated with PI or SB, as similarly found in other studies [25, 42]. As confirmed by Peltzer et al. [25], a positive association was found between being hungry (because there was not enough food in home) and PI among boys. In our study, a girl having an illiterate father was less inactive. Parental education, family income, and even hunger are indicators used to evaluate socioeconomic status of adolescents [25, 44]. Our results, from these three indicators, were different, and, as reported by Mielke et al. [44], it is possible that these different indicators influence health behaviors differently.

There is emerging evidence to link increased PI and SB with many negative psychological indicators among adolescents [11, 18, 23, 28, 29, 45]. We found a positive association between suicidal ideation and SB in girls and boys in our bivariate regression analyses and found loneliness to be positively associated with SB, although only among boys. In the multivariate regression analysis, however, suicidal ideation was negatively associated with PI in boys, but generally, it was still less frequent among physically active adolescents (19.7%). Evidence suggests that physical activity is associated with reduced risk of suicidal thoughts, plans, and attempts [29, 46].

Behavioral factors are the second most studied associated factors of PI and SB in LMIC [20]. Inadequate vegetable intake and no attendance of physical education classes were positively associated with PI in boys. Absenteeism was a risk factor for SB among boys and girls. These associations were confirmed by Peltzer et al. [25]. Unlike previous studies [11, 23, 25, 26], our results did not conclude that adolescents who smoke and consume illicit drug or alcohol tend to be physically inactive and spend more time in sedentary activities.

We found social support, especially parental support and peer support, were significantly and negatively associated with PI and SB [11, 18, 23]. Among girls, in bivariate regression analysis, being bullied or not having enough parental supervision were positively associated with SB and PI, respectively. In a recent meta-analysis, Laird et al. [30] suggested that social support is not a strong predictor of PI in adolescent girls, although parents and friends may have a role in enhancing physical activity. Developing adolescent PI and SB interventions to include social support components could be a promising option.

This study has several limitations. First, assessing PI and SB through self-reporting in pediatric populations may overestimate true physical activity levels, as indicated in a systematic review [47]. Second, it was difficult to compare our results with those from many other studies, as the measure and cut-offs used vary across different studies. Nevertheless, most of time, we compared our results to data generated using the same measure. Environmental factors related to PI and SB were not assessed in this quantitative study but are planned in the qualitative study [36]. Information on menarche (girls only) and past and/or current history of disease can be barriers for the practice of regular physical activity. Unfortunately, information on these covariates has not been collected. Finally, private schools were not included in this study because of their limited numbers in Taza, Morocco.

## Conclusions

In summary, this study represents the first attempt to investigate the factors associated with PI and SB among a sample of Moroccan adolescents. The findings indicate high prevalences of PI and SB, especially among girls. Thus, there is an urgent need to implement appropriate interventions to reduce PI and SB levels in secondary school-age children. Overall, PI and SB were associated with older age, having an illiterate father, being hunger, having suicidal ideation, consuming less vegetables, not attending physical education, and being absent. These results can be useful when designing a future intervention in school-age adolescents.

## Additional files

Additional file 1: Table S1. Description of independent variables^a^. (16.5 Kb)

## Abbreviations

CDC: Centers for Disease Control and Prevention
GSHS: Global School-based Student Health Survey
LMIC: Low- and middle-income countries
PI: Physical inactivity
SB: Sedentary behavior
UNAIDS: United Nations Joint Programme on HIV/AIDS
UNESCO: United Nations Educational, Scientific and Cultural Organization
UNICEF: United Nations Children’s Fund
WHO: World Health Organization

## References

[1] Viner RM, Ozer EM, Denny S, Marmot M, Resnick M, Fatusi A (2012). Adolescence and the social determinants of health. Lancet.

[2] Itoh H, Kitamura F, Hagi N, Mashiko T, Matsukawa T, Yokoyama K (2017). Leisure-time physical activity in youth as a predictor of adult leisure physical activity among Japanese workers: a cross-sectional study. Environ Health Prev Med.

[3] Patton GC, Coffey C, Cappa C, Currie D, Riley L, Gore F (2012). Health of the world’s adolescents: a synthesis of internationally comparable data. Lancet.

[4] World Health Organization. Global health risks: mortality and burden of disease attributable to selected major risks. World Health Organization; 2009.

[5] Kohl HW, Craig CL, Lambert EV, Inoue S, Alkandari JR, Leetongin G (2012). The pandemic of physical inactivity: global action for public health. Lancet.

[6] World Health Organization. Global recommendations on physical activity for health. World Health Organization 2010. 26180873

[7] Janssen I, Leblanc AG (2010). Systematic review of the health benefits of physical activity and fitness in school-aged children and youth. Int J Behav Nutr Phys Act.

[8] Hancock J, Inchley J. Sedentary behaviour. HBSC’s International Coordinating Centre.

[9] American Academy of Pediatrics Committee on Public Education (2001). Children, adolescents, and television. Pediatrics.

[10] Arundell L, Fletcher E, Salmon J, Veitch J, Hinkley T (2016). A systematic review of the prevalence of sedentary behavior during the after-school period among children aged 5–18 years. Int J Behav Nutr Phys Act.

[11] Van Der Horst K, Paw MJ, Twisk JW, Van Mechelen W (2007). A brief review on correlates of physical activity and sedentariness in youth. Med Sci Sports Exerc.

[12] Guthold R, Cowan MJ, Autenrieth CS, Kann L, Riley LM (2010). Physical activity and sedentary behavior among schoolchildren: a 34-country comparison. J Pediatr.

[13] Uijtdewilligen L, Nauta J, Singh AS, van Mechelen W, Twisk JW, van der Horst K (2011). Determinants of physical activity and sedentary behaviour in young people: a review and quality synthesis of prospective studies. Br J Sports Med.

[14] Thorp AA, Owen N, Neuhaus M, Dunstan DW (2011). Sedentary behaviors and subsequent health outcomes in adults a systematic review of longitudinal studies, 1996–2011. Am J Prev Med.

[15] Carson V, Hunter S, Kuzik N, Gray CE, Poitras VJ, Chaput JP (2016). Systematic review of sedentary behaviour and health indicators in school-aged children and youth: an update. Appl Physiol Nutr Metab.

[16] Must A, Tybor DJ (2005). Physical activity and sedentary behavior: a review of longitudinal studies of weight and adiposity in youth. Int J Obes (Lond).

[17] Lee IM, Shiroma EJ, Lobelo F, Puska P, Blair SN, Katzmarzyk PT (2012). Effect of physical inactivity on major non-communicable diseases worldwide: an analysis of burden of disease and life expectancy. Lancet.

[18] Sallis JF, Prochaska JJ, Taylor WC (2000). A review of correlates of physical activity of children and adolescents. Med Sci Sports Exerc.

[19] Atkin AJ, van Sluijs EM, Dollman J, Taylor WC, Stanley RM (2016). Identifying correlates and determinants of physical activity in youth: how can we advance the field?. Prev Med.

[20] Bauman AE, Reis RS, Sallis JF, Wells JC, Loos RJ, Martin BW (2012). Correlates of physical activity: why are some people physically active and others not?. Lancet.

[21] Sallis JF, Cervero RB, Ascher W, Henderson KA, Kraft MK, Kerr J (2006). An ecological approach to creating active living communities. Annu Rev Public Health.

[22] McMinn AM, van Sluijs EM, Wedderkopp N, Froberg K, Griffin SJ (2008). Sociocultural correlates of physical activity in children and adolescents: findings from the Danish arm of the European Youth Heart study. Pediatr Exerc Sci.

[23] Park H, Kim N (2008). Predicting factors of physical activity in adolescents: a systematic review. Asian Nurs Res (Korean Soc Nurs Sci).

[24] Shuval K, Weissblueth E, Brezis M, Araida A, Dipietro L (2009). Individual and socio-ecological correlates of physical activity among Arab and Jewish college students in Israel. J Phys Act Health.

[25] Peltzer K, Pengpid S (2016). Leisure time physical inactivity and sedentary behaviour and lifestyle correlates among students aged 13–15 in the Association of Southeast Asian Nations (ASEAN) member states, 2007-2013. Int J Environ Res Public Health.

[26] Peltzer K (2010). Leisure time physical activity and sedentary behavior and substance use among in-school adolescents in eight African countries. Int J Behav Med.

[27] de Vet E, de Ridder DT, de Wit JB (2011). Environmental correlates of physical activity and dietary behaviours among young people: a systematic review of reviews. Obes Rev.

[28] Asare M, Danquah SA (2015). The relationship between physical activity, sedentary behaviour and mental health in Ghanaian adolescents. Child Adolesc Psychiatry Ment Health.

[29] Hoare E, Milton K, Foster C, Allender S (2016). The associations between sedentary behaviour and mental health among adolescents: a systematic review. Int J Behav Nutr Phys Act.

[30] Laird Y, Fawkner S, Kelly P, McNamee L, Niven A (2016). The role of social support on physical activity behaviour in adolescent girls: a systematic review and meta-analysis. Int J Behav Nutr Phys Act.

[31] Benjelloun S (2002). Nutrition transition in Morocco. Public Health Nutr.

[32] Obermeyer CM, Bott S, Sassine AJ (2015). Arab adolescents: health, gender, and social context. J Adolesc Health.

[33] CDC. Global School-based Health Survey. Available at: https://www.cdc.gov/gshs/countries/eastmediter/morocco.htm.Accessed 01 Jan 2016.

[34] Hamrani A, Mehdad S, El Kari K, El Hamdouchi A, El Menchawy I, Belghiti H (2015). Physical activity and dietary habits among Moroccan adolescents. Public Health Nutr.

[35] High Commission for Planning (HCP). Available at: http://www.hcp.ma/. Accessed 01 January 2016.

[36] El Achhab Y, El Ammari A, El Kazdouh H, Najdi A, Berraho M, Tachfouti N (2016). Health risk behaviours amongst school adolescents: protocol for a mixed methods study. BMC Public Health.

[37] Ministry of National Education, Vocational training, Higher Education and Scientific Research. Available at: http://www.men.gov.ma/En/Pages/Accueil.aspx. Accessed 01 January 2016.

[38] World Health Organization. Global School-based Student Health Survey (GSHS). Available at: http://www.who.int/chp/gshs/en. Accessed 01 January 2016.

[39] Al Subhi LK, Bose S, Al Ani MF (2015). Prevalence of physically active and sedentary adolescents in 10 Eastern Mediterranean countries and its relation with age, sex, and body mass index. J Phys Act Health.

[40] Caleyachetty R, Echouffo-Tcheugui JB, Tait CA, Schilsky S, Forrester T, Kengne AP (2015). Prevalence of behavioural risk factors for cardiovascular disease in adolescents in low-income and middle-income countries: an individual participant data meta-analysis. Lancet Diabetes Endocrinol.

[41] Rahim HFA, Sibai A, Khader Y, Hwalla N, Fadhil I, Alsiyabi H (2014). Non-communicable diseases in the Arab world. Lancet.

[42] Shokrvash B, Majlessi F, Montazeri A, Nedjat S, Rahimi A, Djazayeri A (2013). Correlates of physical activity in adolescence: a study from a developing country. Glob Health Action.

[43] Pearson N, Haycraft E, Johnston JP, Atkin AJ (2016). Sedentary behaviour across the primary-secondary school transition: a systematic review. Prev Med.

[44] Mielke GI, Brown WJ, Nunes BP, Silva IC, Hallal PC (2017). Socioeconomic correlates of sedentary behavior in adolescents: systematic review and meta-analysis. Sports Med.

[45] Hyakutake A, Kamijo T, Misawa Y, Washizuka S, Inaba Y, Tsukahara T (2016). Cross-sectional observation of the relationship of depressive symptoms with lifestyles and parents’ status among Japanese junior high school students. Environ Health Prev Med.

[46] Southerland JL, Zheng S, Dula M, Cao Y, Slawson DL (2016). Relationship between physical activity and suicidal behaviors among 65,182 middle school students. J Phys Act Health.

[47] Adamo KB, Prince SA, Tricco AC, Connor-Gorber S, Tremblay M (2009). A comparison of indirect versus direct measures for assessing physical activity in the pediatric population: a systematic review. Int J Pediatr Obes.

