# Supplementary material for: Level and potential social-ecological factors associated with physical inactivity and sedentary behavior among Moroccan school-age adolescents: a cross-sectional study
Source: Environ Health Prev Med. 2017 May 18;22:47. doi: 10.1186/s12199-017-0657-0 (PMC5664796; doi:10.1186/s12199-017-0657-0)
Supplement: Supplementary file 1 — Description of independent variablesa. (16.5 Kb) [file 12199_2017_657_MOESM1_ESM.docx]

#### **Additional file 1. Description of independent variables ^a^**

| Independent variable | Formulating question | Possible response and codes ^b^ |
| --- | --- | --- |
| Demographic/biological |  |  |
| Gender | Self-reported by students | *Coded 1= boys and 0= girls* |
| Age |  | *Coded 1= 16-17 years, 0= 14-15 years and 2= 18-19 years* |
| Education level of parents |  | *Coded 1= non-educated and 0= educated* |
| Perceived family income  level | How do you describe the economic situation of your family? | 1= good to 3= low *(coded 1= Lower, 0= average and 2= higher)* |
| Hunger | During the past 30 days, how often did you go hungry because there was not enough food in your home? | 1= never to 5= always *(coded 1=sometimes/mostly/always and 0= never/rarely)* |
| Psychological |  |  |
| Loneliness | During the past 12 months, how often have you felt lonely? | 1= never to 5= always *(coded 1= mostly/always and 0= never/rarely/sometimes)* |
| Anxiety | During the past 12 months, how often have you been so worried about something that you could not sleep at night? | 1= never to 5= always *(coded 1= mostly/always and 0= never/rarely/sometimes)* |
| Suicidal ideation | During the past 12 months, did you ever seriously consider attempting suicide? | 1= Yes to 2= No *(coded 1= Yes and 0= No)* |
| Close friends | How many close friends do you have? | 1= 0 or 4= 3 or more *(coded 1= 0 friend and 0= 1 friend or more)* |
| Behavioral |  |  |
| Breakfast intake | During the past 30 days, how often did you eat breakfast? | 1= never to 5= always *(coded 1= never/rarely and 0= sometimes/mostly/always)* |
| Fruit consumption | During the past 30 days, how many times per day did you usually eat fruit, such as bananas, apples, oranges, or any other fruit? | 1= I did not eat fruit during the past 30 days to 7 = 5 or more times per day *(coded 1= < 2 times per day and 0=* $\geq$ *2 times per day)* |
| Vegetable consumption | During the past 30 days, how many times per day did you usually eat vegetables, such as potatoes or tomatoes? | 1= I did not eat vegetables during the past 30 days to 7= 5 or more times per day *(coded 1= < 3 times per day and 0=* $\geq$ *3 times per day)* |
| Walking or biking to school | During the past 7 days, on how many days did you walk or ride a bicycle to or from school? | 1= 0 days to 8= 7 days *(coded 1= < 5 days and 0=* $\geq$ *5 days)* |
| Attendance of physical education | During this school year, on how many days did you go to physical education (PE) class each week? | 1= 0 days to 6= 5 or more days *(coded 1= 0 or 1 day and 0= 2 or more days)* |
| Physical fight | During the past 12 months, how many times were you in a physical fight? | 1= 0 times to 8= 12 or more times *(coded 1= 1 or more times and 0= 0 time)* |
| Tobacco use | During the past 30 days, on how many days did you: smoke cigarettes or other form of tobacco? | 1= 0 days to 7= All 30 days *(coded 1= 1 day or more and 0= 0 day)* |
| Alcohol/illicit drug use | How many times did you drink alcohol in your life? | 1= 0 times to 4= 10 or more times *(coded 1= 1 or more times and 0= 0 time)* |
|  | During your life, how many times have you used drugs: sebsi pipe, hashish, hashish edibles, and psychoactive substance? | 1= 0 times to 5= 20 or more times *(coded 1= 1 or more times and 0= 0 time)* |
| School attendance | During the past 30 days, on how many days did you miss classes or school without permission? | 1 = 0 days to 10 or more days *(coded 1= 1 day or more and 0= 0 day)* |
| Social |  |  |
| Peer support in school | During the past 30 days, how often were most of the students in your school kind and helpful? | 1= never to 5= always *(coded 1= never/rarely/sometimes and 0= mostly/always)* |
| Parental supervision | During the past 30 days, how often did your parents or guardians check to see if your homework was done? | 1= never to 5= always *(coded 1= never/rarely/sometimes and 0= mostly/always)* |
| Parental connectivity | During the past 30 days, how often did your parents or guardians understand your problems and worries? | 1= never to 5= always *(coded 1= never/rarely/sometimes and 0= mostly/always)* |
| Parental bonding | During the past 30 days, how often did your parents or guardians really know what you were doing with your free time? | 1= never to 5= always *(coded 1= never/rarely/sometimes and 0= mostly/always)* |
| Bullying | During the past 30 days, on how many days were you bullied? | 1= 0 days to 7= All 30 days *(coded 1= 1 day or more and 0= 0 day)* |

^a:^ Independent variables were from the GSHS and literature.

^b^: Codes were made according GSHS Data User's Guide and Morocco Public Use Codebook. Details on these tools can be accessed at <http://www.who.int/chp/gshs/methodology/en/>
